# Ultrasonic-Assisted *Ginkgo biloba* Leaves Extract as Natural Antioxidant on Oxidative Stability of Oils During Deep-Frying

**DOI:** 10.3390/foods14172958

**Published:** 2025-08-25

**Authors:** Min Kang, Musfirah Zulkurnain

**Affiliations:** 1 School of Health Management, Shanxi Technology and Business University, Longcheng Campus, Taiyuan 030006, China; kangmin19861110@student.usm.my; 2 Food Technology Division, School of Industrial Technology, Universiti Sains Malaysia, Minden 11800, Penang, Malaysia

**Keywords:** *Ginkgo biloba* leaves flavonoids, natural antioxidant, oxidative stability, frying oils

## Abstract

*Ginkgo biloba* leaf flavonoids, while demonstrating antioxidant potential, remain underexplored as natural stabilizers for frying oils under thermo-oxidative stress. This study assessed *Ginkgo biloba* leaf flavonoids efficacy against synthetic tertiary-butylhydroquinone (0.02%). *Ginkgo biloba* leaf flavonoids were extracted via optimized ultrasonic-assisted methods (15 mL/g solvent, 80% ethanol, 45 °C, 120 s). Frying stability in flaxseed and soybean oils over 6 days (24 cycles/day) was evaluated using multi-indicator kinetic analysis. *Ginkgo biloba* leaf flavonoids significantly outperformed tertiary-butylhydroquinone in reducing oxidation markers after 6 days. In flaxseed oil, *Ginkgo biloba* leaf flavonoids reduced acid value (18.4%), peroxide value (33.79%), and polar compounds (52.03%); reductions in soybean oil reached 61.34% for polar compounds. *Ginkgo biloba* leaf flavonoids better preserved γ-tocopherol in flaxseed oil (increased 2.09% retention) and key tocopherols in soybean oil. Critically, it mitigated unsaturated fatty acid losses (flaxseed C18:3: 2.72% higher; soybean C18:2: 4.4% higher) and limited saturated fatty acid increases. Optimized *Ginkgo biloba* leaf flavonoid extraction facilitates its application as a promising natural candidate for high-temperature frying, where its matrix-specific stabilization effect shows potential in commercial functional oil formulations.

## 1. Introduction

*Ginkgo biloba* (*Ginkgo biloba* L.) is a type of deciduous tree belonging to the Ginkgo family, an ancient tree species native to East Asia. Its aerial parts contain terpene trilactones, acylated flavonol glycosides, biflavones, ginkgotides, and ginkgolic acids [1]. Owing to its wide distribution across China with varying geographical environments and climatic characteristics, *Ginkgo biloba* leaf (GBL) products have different active ingredients and degrees of activity, including flavonoids. Ginkgo has been shown to have over a hundred distinct flavonoid structures that exist as aglycones, glycosides, or dimeric forms called bioflavonoids [2]. The most commonly reported flavonoids in *Ginkgo biloba* are isoginkgetin, ginkgetin, amentoflavone, bilobetin and sciadopitysin, sesquojaflavone, podocarpusflavone A, and 5′-methoxybilobetin [3]. Šamec et al. [2] reviewed the presence of 13 biflavonoids that have been reported in ginkgo leaves, of which amentoflavone, bilobetin, sciadopitysin, ginkgetin, and isoginkgetin are the most common. Standardized extracts of Ginkgo leaves typically contain 24% flavonoids and 6% terpene trilactones (TTLs) [4]. Nevertheless, GBL contains a substance called ginkgolic acids (GA), which are allergenic, genotoxic, cytotoxic, neurotoxic, and mutagenic [5]. However, during heating, ginkgolic acid undergoes a dehydrogenation reaction, generating ginkgolide (diterpenoids) and other substances that possess pharmacological properties such as antibacterial, antioxidant, and anti-apoptotic effects [6], thereby reducing the content of ginkgolic acid. Ginkgolide exhibits exceptional thermal stability [7]. The United States, Chinese, and European Pharmacopoeias state that the concentration of ginkgolic acids (GAs) in GBE should be <5 ppm [1,8,9]. However, despite their negative effects, a beneficial pharmacological effect on the human body has also been shown; for example, ginkgolic acid C17:1 showed various antitumor effects [10]. When alkylphenols are used for medical purposes, if the dosage is appropriate, they possess anticancer and antibacterial properties [11]. Therefore, Ginkgo extract has been used as a food additive [12]. The potentially toxic substances in ginkgo can be reduced to safe levels by heating [13], solvent extraction [14], drying [15], and enzymatic fermentation [16]. Ginkgolide A, B, and other components exhibit excellent antioxidant, anti-cancer, anti-obesity, anti-atherosclerotic, and anti-diabetic effects [17]. The decrease in ginkgolic acid after heat treatment of ginkgo nuts is accompanied by a reduction in hydrocyanic acid [18]. The hydrocyanic acid content of ginkgo nuts can be reduced by 72.40% when steamed at 100 °C for 10 min [19]. Ginkgo nuts were steamed at 115 °C for 75 min, which resulted in a 54.5% reduction in ginkgolic acid content [20].

The different active components in ginkgo leaves exhibit different thermal stabilities. Stability studies have shown that ginkgolide in GBL is highly resistant to heat, acidity, and endopeptidase degradation [21]. The extract of *Ginkgo biloba* leaves was introduced as a natural stabilizer into the polyethylene matrix. The effects of *Ginkgo biloba* extract on the thermal oxidative stability, thermal stability, processing stability, mechanical properties, and long-term aging resistance of polyethylene were systematically investigated [22]. Ginkgolic acid, owing to the presence of carboxyl groups in its molecule that are prone to degradation upon heating, undergoes a decarboxylation reaction rapidly when the temperature reaches 250 °C, converting into ginkgolide [7]. Beta-Ginkgotide, a hyperdisulfide-constrained peptide family of approximately 2 kDa, beta-ginkgotides (beta-gB1 and beta-gB2) from *Ginkgo biloba*, is resistant to thermal, chemical, and proteolytic degradation [23]. During the processing of ginkgo seeds, ultrasonic pretreatment technology before infrared drying (at 80 °C) showed a higher total phenolic content and antioxidant activity, with the best preservation effect on flavonoids [24]. Rohn et al. [25] demonstrated that above 180 °C and over 60 min, flavonol in roasting onion undergoes rapid breakdown. They also mentioned that the sugar moiety was rapidly degraded in the flavonol, resulting in different compounds. Red onions were frying with oil at 180 °C for 4 and 8 min, causing an increase in the levels of total polyphenols, flavonoids, and tannins but a decrease in the levels of phenolic acids and antioxidant activity [26]. Six different varieties of onions were heated at temperatures of 80 °C, 100 °C, 120 °C, and 150 °C. Each temperature was maintained for 30 min, and quantitative analysis was conducted with the onion powder at room temperature. These results indicated that the main flavonoid compounds were destroyed at higher temperatures [27].

Many studies have suggested that the efficacy of Chinese herbal extracts is highly correlated with their antioxidant activity. Different extraction methods and solvents have been reported to exhibit different antioxidant activities. Extraction methods for total flavonoids from *Ginkgo biloba* leaves (GBLF) include solvent, supercritical fluid [28], ultrasonic [29], microwave, and enzymatic extraction [30]. Enhancing the overall yield of bioactive compounds from phytochemical extracts remains a considerable challenge. Traditional solvent extraction methods often require large quantities of botanical materials and organic solvents, are time-consuming, and yield low extraction efficiency. In contrast, ultrasonic-assisted extraction is a rapid, cost-effective, and efficient approach for the extraction of natural active compounds, including GBLF. Moreover, it is more economical and simpler than microwave, supercritical fluid, and enzymatic. Among the identified phenolic compounds, quercetin, kaempferol, and caffeic acid were the predominant phenolic compounds in *Ginkgo biloba*, whereas carnosic acid, rosmarinic acid, narinigen, and hispidulin were the predominant phenolic compounds in rosmarinus officinalis leaves [31].

Ethanol is a widely used solvent for bioactive compound extraction and is relatively safe for humans [32]. Zhang et al. [33] reviewed that by fine-tuning the extraction temperature, it was studied that subcritical ethanol extraction, as a green technology, improved the yield and antioxidant potential of bioactive compounds in sandalwood essential oil. Gong et al. [34] extracted GBLF using graphene oxete-assisted ethanol reflux extraction technology, and the equilibrium yield was 77.03% higher than that of the conventional ethanol reflux extraction method (82.63% vs. 5.6%). The extraction rate and antioxidant activity of flavonoids extracted from bitter gourd fruits using the ethanol-modified SC-CO_2_ extraction method were higher than those obtained using the conventional solvent extraction method [35]. Chen et al. [36] studied the optimal process for extracting flavonoids from cotton fibers. Among them, ultrasonic temperature had the greatest influence on the extraction efficiency, followed by ethanol concentration. Ultrasonic-assisted and ethanol reflux methods were also applied to Astragalus membranaceus [37]. The ultrasonic extraction method refers to the resonance phenomenon and cavitation when an ultrasonic wave is used to act on the extracted substance, causing the breakdown of the cell wall and stronger solvent penetration to dissolve organic components. It has been well documented that ultrasound-assisted extraction increases the extraction rate of flavonoids, including bilobetin (BIL), ginkgetin (GIN), isoginkgetin (IGIN), and sciadopitysin (SCI) [38,39,40]. Characterized by its operation at relatively low temperatures, this method is particularly advantageous for preserving the integrity of bioactive compounds in plant materials [41].

Vegetable oil is a vital component of the human diet, serving not only as a cooking medium with sensory appeal but also as a source of energy, and it plays a crucial role in promoting health and preventing diseases. Flaxseed (*Linum usitatissimum*) oil contains 40–50% α-linolenic acid (ω-3), which is rich in phytosterols and tocopherols. These bioactive components possess the capacity to enhance the human immune system, mitigate inflammation, and aid in the prevention of cardiovascular diseases [42]. Conversely, flaxseed, which is abundant in polyunsaturated fatty acids, is susceptible to oxidation when subjected to frying. This process leads to the formation of free fatty acids (FFAs), peroxides, aldehydes, and other compounds that are harmful to health [43].

Overcoming the stability and shelf life of frying oils is a challenge. During deep frying, several physical and chemical changes occur simultaneously, mainly oxidation, polymerization, and hydrolysis reactions, which alter the chemical composition of edible oils during frying [44]. Antioxidants inhibit lipid oxidation in edible fats and oils, thereby extending shelf life and preserving quality. Effective antioxidants must combine safety, cost-efficiency, stability, organoleptic neutrality, and efficacy at low concentrations. Although synthetic antioxidants such as butylated hydroxyanisole (BHA) and butylatedhydroxytoluene (BHT) exhibit thermolability and volatility at frying temperatures, tertiary butylhydroquinone (TBHQ) demonstrates superior thermal stability, enhancing oil durability during frying and prolonging the shelf life of fried food. However, concerns regarding the potential toxicity, bioaccumulation, and metabolic byproducts of BHA, BHT, and TBHQ necessitate the development of natural alternatives. Consequently, identifying non-toxic, naturally derived antioxidants is critical for sustainable food stabilization.

Medicinal plants are regarded as an efficient source of bioactive compounds, such as polyphenols that exhibit potent antioxidant activity [45]. The addition of natural antioxidants from plant extracts to oils has shown potential benefits in slowing the breakdown of oil during frying and extending the shelf life of fried foods. The ethanol extract of *Myrtus* communis leaves improved the oxidative stability of soybean oil containing phenolic compounds [46]. Hwang et al. [47] reviewed that the 0.1% usage of orange extract had stronger activity, which was similar to 0.02% BHT in soybean oil and was similar or slightly stronger than 0.02% BHT in fish oil. The incorporation of rosemary extract in liposomes into oil delays the oxidation process [48]. The antioxidant activity of 2 and 4 mg/mL concentrations of *Avicennia marina* leaf extract in soybean oil was comparable to that of a 0.2 mg/mL concentration of synthetic TBHQ. Therefore, this natural antioxidant can be used to preserve soybean oil [49]. Okhli et al. [50] researched aqueous, ethanolic, and methanolic extracts of citron peel (800 ppm), BHT synthetic antioxidant (200 ppm), and citron peel essential oil (800 ppm) added to sunflower oil. During 5 days of heat treatment, the sunflower oil sample with European guelder bark extract was characterized by the lowest values of acid, peroxide, and anisidine numbers [51]. Therefore, an increasing number of studies on natural antioxidants and synthetic antioxidants have proven that it is possible for natural antioxidants to replace synthetic antioxidants to inhibit the oxidation and deterioration of vegetable oils. The main active components of GBLF are the glycoside derivatives of quercetin, kaempferol, and isorhamnetin. Their antioxidant mechanisms include the direct scavenging of free radicals (neutralizing reactive oxygen and nitrogen through phenolic hydroxyl groups). Regulating antioxidant enzymes (activating the *Nrf2* pathway) [52] regulates the expression of the antioxidant enzyme and reduces reactive oxygen and nitrogen species, contributing to the reduction in lipid peroxidation [53]. Rosmarinic acid, carnosic acid, and carnosol are the main active components of rosemary extract. It exhibited potent scavenging activity toward 2,2′-diphenyl-1-picrylhydrazyl (DPPH) radicals and superoxide anions [54]. The main component of rosemary, carnosic acid, reduces lipid peroxidation, protein carbonylation, and serum nitric oxide levels to inhibit lipopolysaccharide (LPS)-induced oxidation/nitrification stress [55]. From the two comparisons, rosemary performs better in direct free radical scavenging (such as DPPH), whereas GBLF has more advantages in inhibiting lipid peroxidation and long-term antioxidation in vivo.

The aim of the present study was to optimize the ultrasound extraction parameters by understanding the interaction effects between the extraction parameters and their dynamic changes during ultrasound-assisted ethanol extraction of GBLF and its antioxidant activities. The application of GBLF as a natural antioxidant was then evaluated for the oxidative stability of flaxseed and soybean oils during repeated frying cycles compared to the synthetic antioxidant TBHQ. This understanding of the mechanism of natural antioxidants in oil systems will make a beneficial contribution to the shelf life of frying oil and protect the human body from harmful oxidation products of fats and oils.

## 2. Materials and Methods

### 2.1. Materials

GBL was purchased from the Wanmin Pharmacy (Taiyuan, China). Soybean and flaxseed oil were purchased from Yihai Kerry (Shanghai, China). Semi-finished French fries were sourced from Sanquan Foods Co. (Taiyuan, China). Rutin standard, ascorbic acid, 1,1-diphenyl-2-picryl-hydrazyl radical (DPPH), 2,2′-azino-bis(3-ethylbenzothiazoline-6-sulfonic acid) (ABTS), and TBHQ were obtained from Shanghai Yuanye Biotechnology Co., Ltd. (Shanghai, China), and HPLC-grade methanol (HPLC grade) was purchased from VBS Biologic Inc. (New York, NY, USA).

### 2.2. Preparation of Ginkgo biloba Leaves Extract

The rutin reference standard (20 mg) was dissolved in 30% ethanol and volumetrically diluted to 100 mL to obtain a stock solution (0.20 mg/mL). Aliquots (1–6 mL) of the stock solution were transferred to 25 mL volumetric flasks. Sequential additions were performed as follows:1 mL of 5% NaNO_2_ (vortex mixed, reacted for 5 min), 1 mL of 10% Al(NO_3_)_3_ (vortex mixed, reacted for 5 min), and 6 mL of 4% NaOH. The mixtures were diluted to volume with 30% ethanol and incubated for 15 min. A blank control without rutin was prepared identically. Absorbance was measured at 510 nm (triplicate determinations). A standard curve was constructed by plotting absorbance (*Y*-axis) against concentration (*X*-axis, 0.008–0.090 mg/mL). The linear regression equation was:

Y = 10.914X − 0.0025 (R^2^ = 0.9962) demonstrating significant linear correlation (*p* < 0.001)

The GBL was washed, dried at 50 °C for 24 h, ground using a high-speed grinder (Beijing Yongguang Medical Co., Ltd., Beijing, China), and sieved to 0.2 mm. The extraction was carried out according to Cong et al. [29] using ultrasonic treatment with an ultrasound generator (PLS-SLHJ-2600W, Foshan Prubon Ultrasonic Technology Co., Ltd., Foshan, China) at a working power of 200 W and an operating frequency of 40 kHz. Single-factor experiments were performed to investigate the effects of ethanol volume fractions (50–80%), solvent: material ratio (1:5–1:20 g/mL), temperature (30–60 °C), and time (60–120 s) on flavonoid yield. The extract was filtered, centrifuged (4000 rpm for 10 min), and concentrated by rotary evaporation at 50 °C [56]. The GBL extracts were stored at 4 °C until analysis. The GBLF was purified using AB-8 resin, and the flow rate of the sample was 3 BV/h. The concentration of the sample solution was 0.6 mg/mL, the pH was 3, and the sample was adsorbed at 70 mL and desorbed in 100 mL of 60% ethanol solution after the adsorption.

Total flavonoid content was determined as described by Du et al. [57]. The Ginkgo leaf flavonoid gain rate was calculated using Equation (1).

(1) $$\mathrm{Total}\;\mathrm{flavonoids}=\frac{\rho \times V\times N}{m}\times \;100\%$$

where (ρ) is the mass concentration of the total flavonoids, (V) is the extraction liquid accumulation, (N) is the dilution multiple, and m is the quality of dry powder.

### 2.3. Optimization of the Extraction Process by Response Surface Methodology (RSM)

Optimization of the ultrasonic-assisted extraction conditions for flavonoids from GBL was performed using RSM to solve multivariate problems. Based on the preliminary study of the single-factor experiment, the ultrasonic-assisted extraction of GBL was optimized using the Box-Behnken design for maximum total flavonoid content. The extraction parameters of GBL were solvent: material ratio (X_1_, 1:5–1:15 mL/g), ethanol volume fraction (X_2_, 60–80%), extraction temperature (X_3_, 30–50 °C), and ultrasonic time (X_4_, 80–120 s).

### 2.4. Determination of GBL Antioxidant Activity

GBLF was extracted under optimal process conditions, and the extract was centrifuged, concentrated, and gradient diluted with 50% ethanol by volume to prepare solutions with mass concentrations of 0, 25, 50, 100, 150, and 200 μg/mL.

The antioxidant activity of the *Ginkgo biloba* extract was evaluated by determining the clearance ability of DPPH as described by Liu et al. [58], with appropriate modifications. Briefly, 2 mL of 0, 25, 50, 100, 150, and 200 μg/mL total flavonoid extraction solution was added, and 9 mL of 0.1 mmol/L DPPH-ethanol solution was added. The mixed solutions were kept in the dark for 30 min, and the absorbance was measured at 517 nm. The same amount of absolute ethanol was used as the blank control, and the same concentration of vitamin C was used as the control group. The scavenging ratio % was calculated using Equation (2). All measurements were performed in triplicate (*n* = 3), and results are expressed as mean ± standard deviation.

(2) $$\mathrm{DPPH}\;\mathrm{scavenging}\;\mathrm{activity}=1-\frac{A_{i}-A_{j}}{A_{o}}\times 100\%$$

where A_o_ is the absorbance value of ethanol mixed with the DPPH working solution, A_i_ represents the absorbance value obtained when 2 mL of different concentrations of GBLF are added to DPPH, mixed thoroughly, and subjected to a 30-min reaction at room temperature under light protection, and A_j_ is the control, which is the absorbance value measured when the corresponding concentration of GBLF solution is added to ethanol.

Equal volumes of ABTS solution (7 mmol/L) and potassium persulfate (2.4 mmoL/L) were mixed and incubated in the dark for 16 h. Subsequently, the greenish-blue reagent was diluted with d.H_2_O (1:60 *v*/*v*) to obtain a mixture with an absorbance of 0.701 ± 0.01 at 734 nm. Next, 4 mL of the reagent was added to each sample (10 μL) before incubating the mixture for 6 min. The same concentration of vitamin C was used for the control group. The absorbance of the samples and control was measured at 734 nm; the ABTS radical scavenging activity was calculated according to Equation (3). All measurements were performed in triplicate (*n* = 3), and results are expressed as mean ± standard deviation.

(3) $$\mathrm{ABTS}\;\mathrm{scavenging}\;\mathrm{activity}=\frac{A_{1}-A_{2}}{A_{C}}\times 100\%$$

where A_1_ is the absorbance value of solutions containing different concentrations of GBLF and ABTS, A_2_ is the absorbance value of the solution containing ethanol and ABTS+ (without samples), and Ac is the initial absorbance value of the solution containing ABTS+ (without samples and solvent) and represents the maximum absorbance when no samples are added.

### 2.5. Preparation of Frying Oil Protocol

To fully study the antioxidant properties of GBL, flaxseed oil (rich in linolenic acid, 40–60%) and soybean oil (rich in linoleic acid, content 50–65%) with different fatty acid compositions were selected as sample oils. As linolenic acid contains three double bonds and linoleic acid contains two double bonds, the total content of unsaturated fatty acids in flaxseed oil is higher than that in soybean oil, which makes flaxseed oil more prone to oxidation.

To assess the inhibitory effect of GBL extracts on thermo-oxidative lipid degradation, optimal conditions of ultrasonic-assisted solvent extraction and resin purification GBLF were added to flaxseed oil and soybean oil according to the ultrasonic-assisted extraction process optimized by RSM, 0.02% TBHQ was added to the two oil samples, followed by continuous frying without replenishment using a 4 kg domestic fryer (Foshan Siboshi Co., Ltd., Foshan, China) using a digital thermometer to monitor the oil temperature. The oils were preheated to 180 ± 2 °C before frying a batch of 100 g par-fried potato sticks for 5 min. The frying oil was kept at 180 °C for 15 min before the next frying cycle of potato sticks, and the operation was repeated for six consecutive days for 8 h daily for a total of 24 frying cycles per day. Oil samples (100 mL) were collected each day after every 24 frying cycles, filtered in a screw tube using degreased gauze, and stored rapidly at −20 °C for further analyses [59].

### 2.6. Determination of Chemical Property of Frying Oil

#### 2.6.1. Determination of the Acid Value (AV)

The acid value was determined using the official method of AOCS Cd 3d-63. First, an appropriate mass of the sample was weighed and placed in a conical flask, and 50 mL of the ethyl ether-isopropyl alcohol mixture (1:1, *v*/*v*) was added, as well as 3 to 4 drops of the phenolphthalein indicator. The mixture was shaken thoroughly to dissolve the sample and was titrated with a standard potassium hydroxide titration solution. The sample solution first appeared slightly pink, and there was no apparent fading within 15 s. The volume (in mL) of the standard titration solution consumed at this endpoint was recorded; this value is denoted as V. Acid value was calculated according to the following formula:

(4) $$\mathrm{AV}\;=\;\frac{(V-V_{0})\times c\times 56.1}{m}$$

where V and V_0_ represent the volumes of standard titration solution consumed in the sample titration and the blank test (mL), respectively, c is the concentration of the standard titration solution (mol/L), m is the weight of the sample (g), and 56.1 is the molar mass of potassium hydroxide.

#### 2.6.2. Determination of the Peroxide Value (PV)

The peroxide value was determined using the official method of AOCS Cd 8b-90. Generally, 2 g of oxidized oil sample was weighed in a volumetric flask (250 mL), then a 30 mL mixture of chloroform and glacial acetic acid (2:3, *v*/*v*) was added, the flask was shaken, and then 1.0 mL of saturated potassium iodide solution was added. Subsequently, it was plugged immediately, gently shaken for 30 s, and placed in the dark for 3 min, after which 100 mL of distilled water was added. After shaking, the mixture was titrated with standard sodium thiosulfate solution. Then, 1 mL of starch indicator was added when the solution became light yellow, followed by continued titration until the blue color disappeared, which was the end point of the titration. The blank assay was performed under the same conditions. PV was expressed as millimoles of reactive oxygen species in a 1 kg sample (mmol/kg) and calculated according to the following equation:

(5) $$\mathrm{PV}\;=\;\frac{(V-V_{0})\times c}{2\times m}\times 1000$$

where V and V_0_ are the volumes of the sodium thiosulfate standard solution consumed by the sample titration and the blank assay (mL), respectively, c is the concentration of the sodium thiosulfate standard solution, m is the weight of the sample (g), and 1000 is the conversion factor.

#### 2.6.3. Determination of Anisidine Value (p-AV)

The p-AV for all the oil samples was determined according to the AOCS method Cd 18-90. Weigh 0.5 g of the test sample into a 25 mL volumetric flask, dissolved and diluted to volume with isooctane. The absorbance (A_b_) of the solution was measured at 350 nm in a cuvette with a spectrophotometer, using the reference cuvette filled with solvent as a blank. Five milliliters of the oil solution were pipetted into one test tube, and 5 mL of the solvent were placed into a second test tube. Using an automatic pipette, 1 mL of the p-anisidine reagent was added to each tube and shaken. After exactly 10 min, the absorbance (A_s_) of the solvent in the first test tube was measured at 350 nm in a cuvette with a spectrophotometer, using the reference cuvette filled with solvent as a blank. Five milliliters of the fat solution were pipetted into one test tube, and 5 mL of the solvent were placed into a second test tube. By means of an automatic pipette, 1 mL of the p-anisidine reagent was added to each tube and shaken. After exactly 10 min, measure the absorbance (As) of the solvent in the first test tube in a cuvette at 350 nm, using the solution from the second test tube as a blank in the reference cuvette. The p-anisidine value (p-AV) is given by the formula:

(6) $$p\text{-}\mathrm{AV}\;=\;\frac{25\times (1.2\times \mathrm{As}-\mathrm{Ab})}{m}$$

where A_s_ absorbance of the oil solution after the reaction with the p-anisidine reagent, A_b_ is the absorbance of the oil solution, and m is the mass of the test portion, g.

#### 2.6.4. Polar Compounds (PC)

PC was determined by Zhu et al. [60]. PCs in the frying oils were evaluated using an edible oil polar compound (EOPC) flash chromatography system (Tianjin Bonna-Agela Technologies Co., Tianjin, China)) (AOCS Official Method Cd 20-91). Following AOCS Official Method Cd 20-91, non-Polar and polar compounds were eluted with petroleum ether/diethyl ether (250 mL, 87:13, %*v*/*v*) and acetone/diethyl ether (250 mL, 40:60, *v*/*v*), respectively. The eluents were dried using atmospheric pressure rotating evaporation (YaoTe, Shanghai, China) and a vacuum drying oven (YiHeng, Shanghai, China) for 1 h at 45 °C. Finally, the PCs content was calculated based on the weight of nonpolar compounds. All measurements were performed in triplicate (*n* = 3), and results are expressed as mean ± standard deviation.

### 2.7. Determination of Tocopherol Contents of Frying Oil

Oil samples (0.5 g) were dissolved in ethanol, and the oils (2–3 mL) were filtered through a 0.22 μm microfiltration membrane. Each oil sample was independently prepared and analyzed in triplicate (*n* = 3). Tocopherol content was quantified by comparison with external standard calibration curves using HPLC determination (Agilent 1200, Agilent Technologies, Santa Clara, CA, USA). The calibration curves were constructed using authentic α-, γ-, and δ-tocopherol standards (Sigma-Aldrich, St. Louis, MO, USA, purity ≥ 98%) dissolved in ethanol. A series of standard solutions covering the concentration range of 1–50 μg/mL for each tocopherol were analyzed. Linear regression analysis yielded correlation coefficients (R^2^) greater than 0.999 for all analytes. Chromatographic conditions were as follows: column, PFP (250 mm × 4.6 mm, 5 μm particle size); column temperature, 30 °C; mobile phase, methanol-water (93:7, *v*/*v*); flow rate, 0.7 mL/min; excitation wavelength, 294 nm; emission wavelength, 328 nm; and injection volume, 10 μL [61]. Under these conditions, α-, γ-, and δ-tocopherol were separated at the baseline. Identification of tocopherols in the samples was based on matching their retention times (α-tocopherol RT ≈ 6.5 min; γ-tocopherol RT ≈ 9.2 min; δ-tocopherol RT ≈ 12.5 min) with those of the corresponding standards. The tocopherol content for each sample was calculated as the mean of triplicate determinations, and the results are expressed as mean ± standard deviation (SD).

### 2.8. Determination of Fatty Acid Contents of Frying Oil

Fatty acid methyl esters were prepared from 0.5 g oil samples by base-catalyzed transesterification according to AOCS Cd 19-90 (2012). Briefly, the samples were reacted with 0.5 M sodium methoxide in methanol (4 mL) at 50 °C for 1 h, followed by extraction with hexane (2 times with 3 mL each). The combined hexane extracts were washed with distilled water (2 mL) and dried over anhydrous sodium sulfate. Prior to derivatization, non-volatile impurities were removed by centrifugation at 10,000× *g* for 5 min. Fatty acid methyl ester analysis was performed using a Shimadzu GC gas chromatograph equipped with a DEGS capillary column (40 m × 0.25 mm ID, 0.25 μm film thickness). Detector: Flame ionization detector (FID). Gases: Nitrogen carrier (linear velocity 20 cm/s), hydrogen 40 mL/min, and air 400 mL/min. Temperature: Injector 220 °C, detector 240 °C. Oven program: Isothermal at 170 °C for 2 min, then ramped at 4 °C/min to 220 °C (held for 10 min). Injection: 1 μL split injection (50:1 ratio).

Quantification and quality control: Calibration: Six-point calibration curve (0.5–50 mg/mL) using a 37-component FAME mix (CRM47885, MilliporeSigma, Burlington, MA, USA). Linear regression achieved a R^2^ > 0.998 for all major fatty acids (C16:0, C18:0, C18:1, C18:2, and C18:3). Identification: Peak assignment by relative retention time vs. C18:0 methyl ester (per Cd 19-90 Table 1). Replicates: Each sample underwent independent derivatization and GC analysis in triplicate (*n* = 3). Precision: RSD < 3% for major fatty acids (intraday, *n* = 5). Recovery: 92–105% for spiked palmitic acid (C16:0) standard.

### 2.9. Statistical Analyses

The data from the RSM were statistically analyzed using Design Expert version 6.0.10 (Stat-Ease, Inc., Minneapolis, MN, USA). Each model was analyzed using ANOVA by using SPSS 26.0 (IBM Corp., Armonk, NY, USA), regression analysis with hierarchical backward elimination at a 95% significance level (*p* < 0.05), and response surface plotting to establish the optimum conditions for the ultrasonic extraction of *Ginkgo biloba* leaves. Insignificant (*p* > 0.05) factors and interactions between them were removed. The data from each response were fitted using multiple regressions with a second-order polynomial model equation.

## 3. Results and Discussion

### 3.1. Ultrasonic Assisted Extraction of Flavonoid from GBL

GBL, which comprises many phytoconstituents, terpene triesters (ginkgolides), and flavonoid compounds, has the most attention in the research on ginkgo so far [1,62]. Gong et al. [63] reported that the most representative flavonoid in ginkgo leaves, isorhamnetin, has functions such as protection of cardiovascular and cerebrovascular systems, anti-tumor, anti-inflammatory, antioxidant, organ protection, and prevention of obesity. *Ginkgo biloba* leaves also contain a toxic substance named ginkgolic acid (GA), but the heating process may cause ginkgolic acid to undergo dehydrogenation reactions, thereby decomposing it into ginkgolide, which has high biological activity and reduces the toxicity of ginkgolic acid [5]. Selecting the correct solvent that matches the polarity of flavonoid compounds maximizes their extraction efficiency because flavonoids are soluble in polar solvents, such as ethanol, water, and acetone. Also, applying ultrasonic treatment during solvent extraction involves acoustic cavitation, which can influence solvent penetration, cell wall disruption, and subsequent release of intracellular compounds but has the potential to destroy flavonoid compounds due to localized high temperatures and pressures created when microbubbles collapse during cavitation. Thus, optimization of the ultrasound extraction parameters was conducted using RSM to understand the interaction effects between the combined effects of ultrasound and ethanol extraction of GBLF. The influencing factors were the solvent: material ratio (X_1_), ethanol volume fraction (X_2_), extraction temperature (X_3_), ultrasonic time (X_4_), and extraction rate of flavonoids as the response value. Response surface optimization experiments were performed to determine the influencing factors. The variance analysis of the reduced quadratic model of the total flavonoid content is presented in Table 2, where the adequacy and fitness of the reduced model were sufficiently explained by the coefficient of determination, R^2^(0.9790), and the non-significant lack-of-fit test (*p* = 0.07).

**Figure 1:**
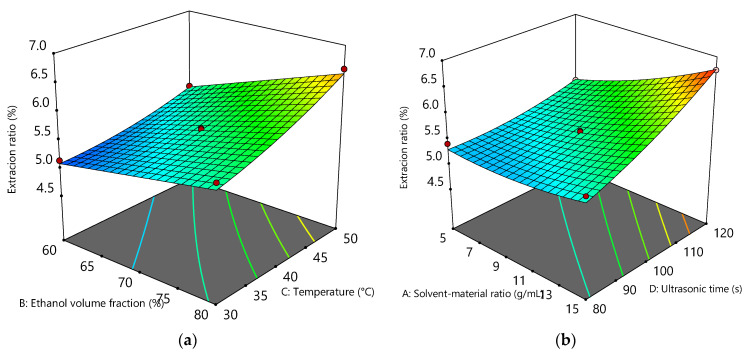
Response surface plots of total flavonoid content of *Ginkgo biloba* leaf extracts showing the interaction between (**a**) solvent:material ratio and ultrasonic time; and (**b**) ethanol volume fraction and extraction temperature. The color gradient ranges from blue to red, corresponding to the increase in predicted yield.

Ultrasonic ethanol extraction (Table 3) showed the strongest influence from ultrasonic time (F-value = 188.84), followed by ethanol volume fraction (X_2_) (F-value = 155.01), extraction temperature (X_3_) (F-value = 137.77), and solvent: material ratio (X_1_) (F-value = 123.55) with significant quadratic and interaction effects. Longer ultrasonic exposure enhanced solvent penetration and cell wall disruption from the generation of acoustic cavitation, which facilitated the subsequent release of intracellular compounds such as flavonoids (7.08%). In this case, the polarity of ethanol is important to match the polarity of most Ginkgo flavonoids for maximum extraction. Response surface plots of flavonoid content showing significant interaction effects between solvent, material ratio and extraction time, and ethanol volume and extraction temperature (Figure 1) were generated using a quadratic polynomial equation (coded factors) as follows:

Y = 5.6 + 0.33X_1_ + 0.37X_2_ + 0.35X_3_ + 0.40X_4_ + 0.23X_1_X_4_ + 0.12X_2_X_3_ + 0.10X_1_^2^ + 0.08X_3_^2^ + 0.11X_4_^2^.

The interaction between solvent:material ratio and ultrasonic time was the most pronounced (*p* < 0.05). An increase in ultrasonic time resulted in the amplification of the flavonoid extraction rate with an increase in the solvent:material ratio up to 6.8%. The increased ultrasonic time can enhance the ultrasonic reaction with the sample and accelerate solvent and energy penetration into the matrix [64]. The interaction between solvent:material ratio and ultrasonic time was significant (*p* < 0.05). The ethanol volume fraction showed amplification of the flavonoid extraction with an increase in the extraction temperature (Figure 1). The increase in extraction temperature could enhance the flavonoid dissolving power because high temperatures can speed up the movement of the solute molecules [65]. Ethanol at different polarities can solubilize flavonoid glycosides owing to their intermediate polarity, which can be tailored to the polarity of most flavonoid compounds such as kaempferol, quercetin, and isorhamnetin [18]. A higher extraction temperature requires a volume fraction of ethanol for maximum flavonoid extraction. When the concentration of ionic liquid increased from 0.025 mol/L to 0.15 mol/L, the extraction efficiency of bilobetin (BIL), ginkgetin (GIN), isoginkgetin (IGIN), and sciadopitysin (SCI) gradually increased [38].

According to the results and discussion, the optimum extraction was required to find the desired condition for maximizing the extraction efficiency of GBLF, solvent: material ratio 14.93 mL/g, ethanol volume fraction 79.38%, extraction temperature 46.02 °C, and ultrasonic time 113.58 s. Extracted 2 times according to the above condition, the maximum extraction efficiency of GBLF was 7.08 ± 0.31%, and the predicted maximum extraction efficiency fitted by the software was 7.33%, which correlated quite well with the actual data, demonstrating that the model could simulate the reality and the optimum conditions were quite valid for this experiment. GBLF was purified using AB-8 resin, and the flow rate of the sample was 3 BV/h. The concentration of the sample solution was 0.6 mg/mL, the pH was 3, the sample was adsorbed at 70 mL, and it was desorbed in 100 mL of 60% ethanol solution after adsorption. The purity of the purified samples increased from 7.08% to 36.20% and was added to the frying oil in the next experiment. Quercetin, Kaempferol, Rutin, Quercetin 3-glucoside, Isorhamnetin, Catechin, Apigenin, Luteolin 7-O-glucoside, Luteolin, Naringin, Naringenin, Gallocatechin, Epigallocatechin, and Myricetin were identified by LC-MS in GBL [66].

### 3.2. Antioxidant Activity of Extract

**Figure 2:**
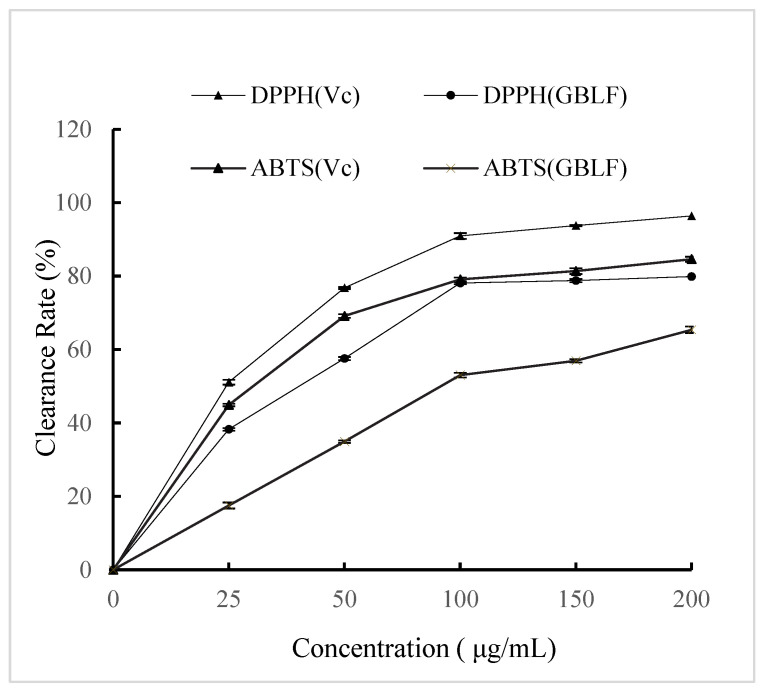
DPPH and ABTS values of optimized Ginkgo biloba leave extracts compared to ascorbic acid standard (Vc).

Flavonoid compounds are of great importance as natural antioxidants in vegetable oils because they protect them against oxidative damage. Natural antioxidants are primarily plant phenolics derived from different plant parts. The effectiveness of flavonoids in retarding lipid oxidation in fat-containing foods is related to their ability to act as free-radical acceptors or chelators of metal ions [67,68]. Figure 2 shows that the DPPH scavenging ability of GBLF was slightly lower than that of vitamin C (*p* < 0.05), showing a similar scavenging ability pattern [69]. Chen et al. [70] reviewed the DPPH radical scavenging activity increase with the GBL polysaccharide concentration; this increase became less obvious when the concentration exceeded 2 mg/mL, whereas the overall DPPH radical scavenging activity was not as strong as ascorbic acid. When the concentration of *Ginkgo biloba* flavonoids was 200 μg/mL, the DPPH scavenging rate was 79.88 ± 0.42%, whereas at the same concentration, the rate of vitamin C was 96.39 ± 0.18%. The same situation was also observed for the ABTS clearance rate. At a concentration of 200 μg/mL, the GBLF clearance rate was 19.23% lower than that of vitamin C. Kuncoro et al. [71] reviewed flavonoid compounds of Krokot Herb (Lygodium microphyllum) and showed high antioxidant activity against DPPH. The antioxidant activity of flavonoids is generally governed by their chemical structure. Extensive hydroxylation of flavonoid compounds in *Ginkgo biloba* leaves enhances their antioxidative effects. The high antioxidant capacity values found in blueberry extracts might be attributed to their high content of total polyphenols, flavonoids, and anthocyanins, which showed higher DPPH [72].

Previous studies have similarly shown a linear correlation between vitamin C equivalent antioxidant capacity, as measured by the ABTS radical assay, and increasing concentrations of antioxidant phenolics [73]. The total flavonoid content of different parts of Alhagi pseudalhagi was in the order of leaf > stem > root, and the total flavonoid content of different parts had strong scavenging effects on DPPH and ABTS radicals [74]. Similar antioxidant properties have also been discovered in the seeds of ginkgo trees [15] and *Ginkgo biloba* dark tea [13].

### 3.3. Changes of Frying Oils Oxidative Stability During Repeated Frying Cycles

Depending on the structural features of the antioxidative compounds, the mechanism of action may include: scavenging free radicals, chelating prooxidant metals, quenching singlet oxygen, inactivating sensitizers, creating oxygen barriers, and decomposing or removing lipid degradation products. However, mechanisms by which antioxidant functions may vary at different frying stages.

#### 3.3.1. Acid Value

**Figure 3:**
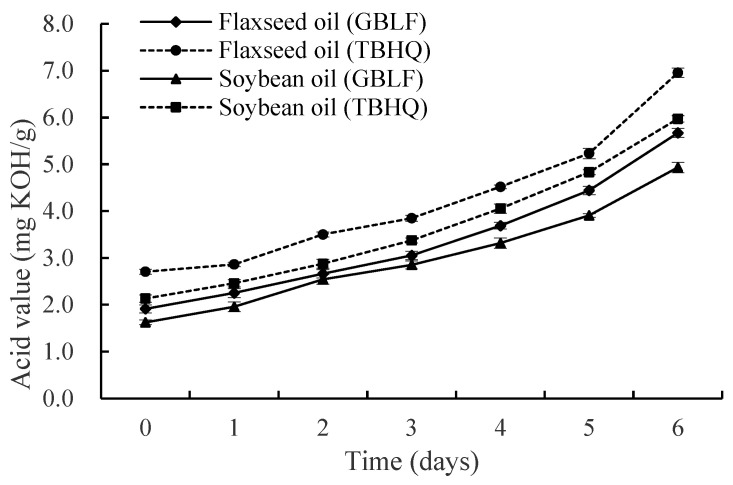
Acid value of flaxseed and soybean oils treated with *Ginkgo biloba* leaf extracts compared to synthetic antioxidants during deep frying.

The acid value (AV) reflects free acids in oils, including the free fatty acids (FFAs) resulting from oil hydrolysis. The acidity of the oil gradually increased until the end of the experiment because of the hydrolysis of triglycerides into FFAs, the transformation of the secondary oxidation products to free acids, and the loss of the ability of the extract to inhibit hydrolysis. Changes in the acid values of the treated oils as a function of time are shown in Figure 3. Flaxseed oil treated with GBLF exhibited a mean lower AV of 21.03% compared to the commercial antioxidant TBHQ at 0.02% throughout the frying process (*p* < 0.05). Soybean oil treated with *Ginkgo biloba* flavonoid showed the lowest AV of 17.97% with lower AV for soybean oil treated with TBHQ (*p* < 0.05). The *Ginkgo biloba* flavonoid extract was more effective in inhibiting lipid oxidation during thermal treatments in soybean oil than in flaxseed oil, although high concentrations were needed compared to TBHQ. These results show that GBLF protects the oxidative stability of oils and is more effective in protecting flaxseed oil, which may be due to the different fatty acid composition of the two oils. Our results agree with those of Zheng et al. [75], who reported that the AV of fried loach oil reached 2.46 mg/g after nine frying cycles. Silva et al. [76] evaluated the physicochemical parameters and demonstrated that the formation of degradation products in the thermochemical reactions occurring during the continuous frying of partially hydrogenated soybean oil and refined palm oil was associated with an increase in the acidity index, peroxide index, and aniline value of both oils. The inhibition of the acid value increase may suggest that flavonoids reduce triglyceride hydrolysis by suppressing lipase activity. However, in a high-temperature frying environment, enzyme activity is usually low; therefore, metal chelation may simultaneously and indirectly reduce the hydrolysis reaction catalyzed by metal ions. Song et al. [22] reported the addition of GBL improved the thermal oxidation stability and long-term aging resistance of the PE matrix even after multiple extrusions.

#### 3.3.2. Peroxide Value

**Figure 4:**
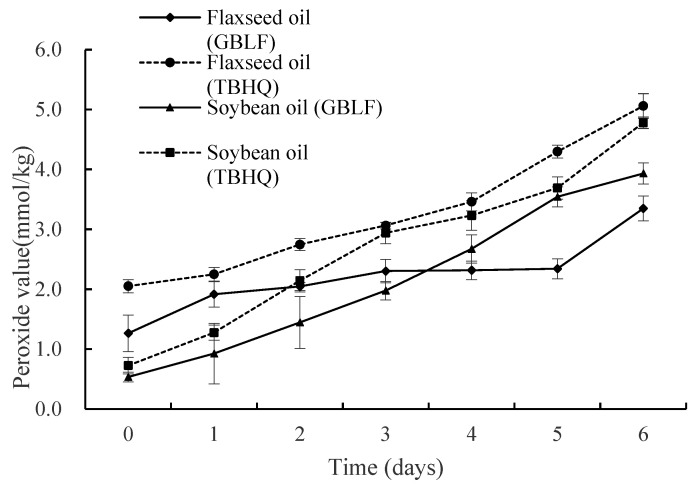
Peroxide value of flaxseed and soybean oils treated with *Ginkgo biloba* leaf extracts compared to synthetic antioxidants during deep frying.

The influence of oils treated with GBLF and TBHQ on peroxide value (PV) is shown in Figure 4. PV measures dissolved oxygen in oil and reflects the amount of hydroperoxide generated during the initial stages of lipid oxidation and the oil quality [77]. The peroxide value can be used to indicate the amount of hydroperoxides generated by rancidity and to characterize the extent to which antioxidants inhibit these oxidation products that occur as a result of auto-oxidation of the oil [78]. In flaxseed oil containing GBLF and TBHQ, PV exhibited a mean decrease of 30.82% compared to TBHQ at 0.02% throughout the frying process (*p* < 0.05). Soybean oil treated with GBLF showed a lower PV (22.54%) than soybean oil treated with TBHQ (*p* < 0.05). Soybean oil treated with GBLF showed the lowest PV at 3 days or less, compared with TBHQ. However, flaxseed oil with GBLF showed the lowest PV between 4 and 6 days of frying. Soybean oil was more effective in inhibiting lipid oxidation during thermal treatment in the early stage of frying, and flaxseed oil was more effective in the later stage of frying.

A continuous increase in PV with increasing frying time was observed for all samples, but the PV of the oils treated with GBLF was lower than that of the TBHQ control. This indicates that GBLF has a significant effect on the PV of oil during frying and can significantly inhibit the formation of hydroperoxide during frying better than TBHQ. In the last 6 days, the PV rise of flaxseed oil was lower than that of soybean oil, which may be due to the unstable hydroperoxides generated during frying, which further decomposed into small molecules of aldehydes, ketones, and acids. Compared with soybean oil, the decomposition rate of hydroperoxides after frying flaxseed oil is faster, and when the decomposition rate is greater than the generation rate, the peroxide value is reduced. The PV of fried loach was unqualified after 9 and 4 frying (19.17 meq O2/kg) [75]. The content of peroxide reflects the accumulation of primary oxidation products (hydroperoxides) and is directly related to the free radical chain reaction. Experiments have shown that flavonoids provide hydrogen atoms through phenolic hydroxyl groups, neutralize lipid peroxyl radicals (*LOO·*), and block oxidation chain reactions [79]. Flavonoids can chelate the iron ions and copper ions present in flaxseed oil and soybean oil, inhibit the catalytic decomposition of hydroperoxides into free radicals, and indirectly reduce the peroxide value.

#### 3.3.3. Anisidine Value

**Figure 5:**
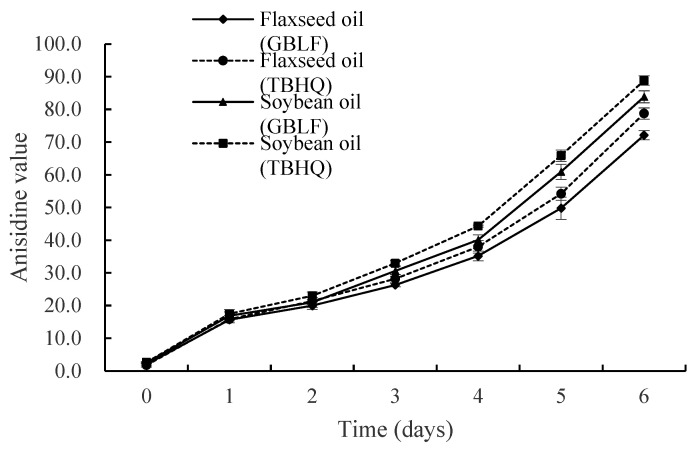
Anisidine value of flaxseed and soybean oils treated with *Ginkgo biloba* leaf extracts compared to synthetic antioxidants during deep frying.

Hydroperoxides rapidly decompose into stable alpha and beta unsaturated aldehydes and ketones during frying, deteriorating the flavor and odor of the oil [80]. GBLF inhibited secondary oxidation product formation more efficiently than TBHQ (*p* < 0.05) (Figure 5), with an 8.42% reduction in the anisidine value (p-AV) on day 6 in flaxseed oil. Compared to soybean oil, GBLF treated with frying oil also showed lower antioxidant activity, whereas in soybean oil, GBLF addition resulted in a 5.57% reduction in p-AV on day 6 compared to that of TBHQ. As expected, GBLF significantly inhibited the formation of secondary products compared with TBHQ, in which aldehydes and ketones started to accumulate slowly during frying. In 6 days, the anisidine value of soybean oil was higher by 10.07 and 11.75 than those of flaxseed oil in TBHQ and extraction treatments, respectively (*p* < 0.05), which suggests that more secondary oxidation products were produced after frying in soybean oil [81,82]. This represents the stage of secondary oxidation product formation. In the experiment, *Ginkgo biloba* extract reduced the anisidine value in oils to a greater extent than TBHQ, indicating that flavonoids indirectly inhibit the formation of secondary products by reducing the accumulation of primary oxidation products. This is the extended effect of free radical scavenging ability.

#### 3.3.4. Polar Compounds

**Figure 6:**
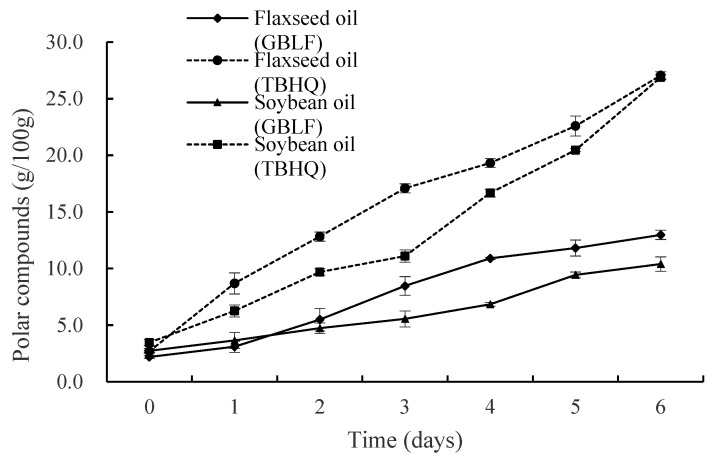
Polar compounds of flaxseed and soybean oils treated with *Ginkgo biloba* leaf extracts compared to synthetic antioxidants during deep frying.

Polar compounds (PC) consist of triglyceride dimer (TGD), triglyceride oligomer (TGO), oxidized triglyceride monomer (oxTGMs), diglyceride (DG), and free fatty acid (FFA), which comprehensively reflect the total amount of oxidation and hydrolysis products. Figure 6 shows that the PC of oils treated with synthetic antioxidants increased more rapidly during repeated frying cycles for both flaxseed and soybean oils at 27.1% and 26.9%, respectively, after six days. These PC values exceeded the maximum level of frying polar substances specified in Germany (24%), Belgium, France, Portugal, Italy, and Spain [83]. In contrast, the PC contents of flaxseed oil and soybean oil treated with GBLF were 12.98% and 10.40%, respectively (*p* < 0.05), which was significantly lower than that of the control after six days of repeated frying cycles [75]. The antioxidant of GBLF stops the chain reaction rate at different stages of oxidation or captures chain free radicals to prevent chain proliferation, which delays and blocks oxidation, thereby delaying food oxidation. Chen et al. [84] compared the influence of oil type on the polar compound composition of vegetable oils (peanut, rapeseed, soybean, and linseed oils) during different oxidation processes (Schaal oven test, room temperature storage, and traditional Chinese cooking); the influence of oil deterioration on the oxidized and polymeric products was relatively more significant than that of others during oxidation processes. The triglyceride oligomers in flaxseed oil were higher than those in other oils under the same conditions, which could be attributed to the high percentage of polyunsaturated fatty acids in the oils, leading to a high level of oxidized and polymeric triglycerides.

Currently, changes in the content of polar compounds during frying have been extensively studied, and both the type of oil and type of food are factors that influence the formation of polar compounds in frying oils. This is consistent with the results of Elaine et al. [85], who determined the maximum frying cycles of selected canola oil, corn oil, groundnut oil, palm oil, and sunflower oil during intermittent frying of French fries and showed that acid value (AV), peroxide value (PV), p-anisidine value (p-AnV), and TPC increased every 8th interval of the frying cycle. Polar compounds (PCs) in palm oil (PO), rapeseed oil (RO), and high-oleic sunflower oil (HOSO) were monitored in French fries (FF), chicken nuggets (CN), and fish nuggets (FN). Real restaurant deep-frying systems showed oil type, fried food, and the interaction between them significantly (*p* < 0.001) affected the polar compound distribution [86]. The reduction in PC in the experiment might be due to the synergy of multiple mechanisms, involving both free radical scavenging (reducing oxidation products) and metal chelation (inhibiting metal catalytic oxidation) simultaneously. However, enzyme inhibition had a relatively small impact on TPC because the contribution of the hydrolysis reaction to the oxidation process was limited.

#### 3.3.5. Tocopherol Contents

**Figure 7:**
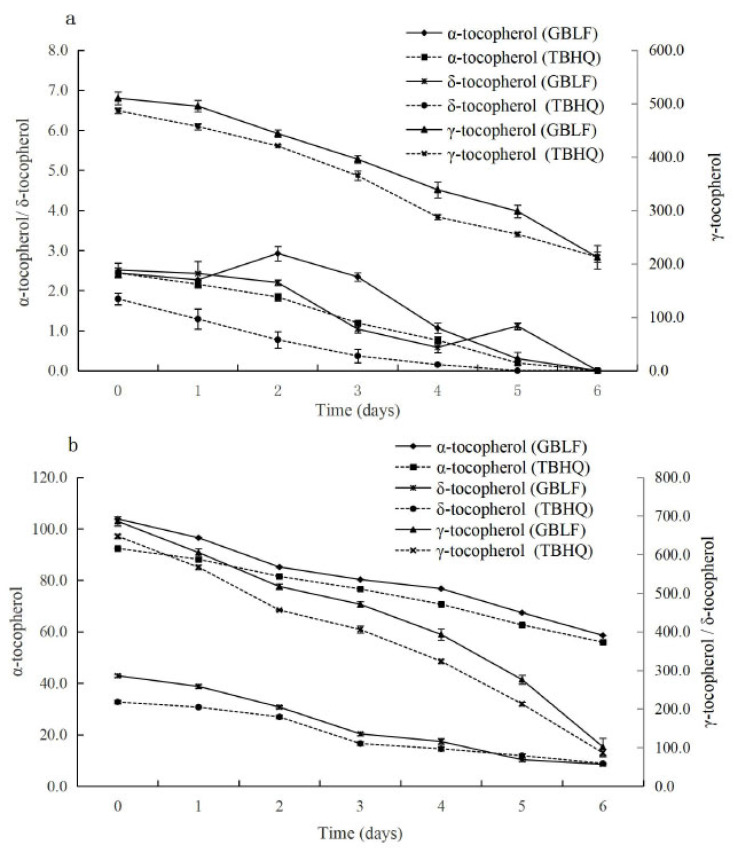
(**a**) The chart depicts the tocopherol contents in flaxseed oil treated with *Ginkgo biloba* leaf extracts compared to synthetic antioxidants during deep frying; (**b**) The figure shows parallel data for soybean oil, illustrating the tocopherol contents when treated with the same extracts and antioxidants under deep frying conditions.

Tocopherols are natural antioxidants in vegetable oils and are the most important nutrients in fats and oils. Tocopherols are the most important nutrients in fats and oils, and they mainly have eight types (α, β, γ, δ, etc.), which have strong antioxidant activities. However, they underwent severe depletion during heating. In Figure 7, the tocopherol content of flaxseed oil and soybean oil was mainly α-tocopherol, γ-tocopherol, and δ-tocopherol. As shown in Figure 7a, the α-tocopherol and δ-tocopherol losses were 100% for flaxseed oil frying for 6 days, and γ-tocopherol decreased by 58.4% with GBLF and decreased by 56.3% with TBHQ (*p* < 0.05). However, during the frying period in soybean oil, α-tocopherol in *Ginkgo biloba* extract decreased by 43.59%, which was 4.18% higher than that of the TBHQ treatment (*p* < 0.05). A similar trend was observed for δ-tocopherol. In Figure 7b the γ-tocopherol content of soybean oil decreased by 85.13% with *Ginkgo biloba* extract, while it decreased by 86.67% with the TBHQ control (*p* < 0.05). The degradation rates of vitamin E and carotenoids depend on the type of fat and oil and are modulated by the processing techniques applied, especially in oil refining and the end use of oil. Mba et al. [87] showed that vitamin E isomers and carotenoids are stable in crude palm oil and blends after 15 h of repeated deep-fat frying at 170 °C and 180 °C.

During frying, tocopherol oxidation was distinct in all the samples. Figure 7 showed a continuous reduction in frying, and the concentration began to decline steadily. This could be related to the depletion and degradation of tocopherols in the native oil due to thermal processing [88]. The tocopherol loss in both flaxseed oil and soybean oil containing GBLF was lower than that in the control TBHQ. The results indicate that GBLF significantly protects against the loss of tocopherol content in flaxseed and soybean oils during frying, compared to TBHQ (*p* < 0.05). This may be attributed to the fact that GBLF contains hydroxyl groups, which provide hydrogen atoms to interrupt the oxidative chain reaction and effectively scavenge reactive oxygen and lipid radicals [87]. Zeb and Nisar [88] studied the carotenoid, chlorophyll, and tocopherol contents of spinach leaves after frying in sunflower oil and found that the increased stability of sunflower oil may be due to the presence of tocopherol in spinach leaves, as well as in sunflower oil. Tocopherols are strong natural antioxidants that stabilize frying oils [89]. Blekas et al. [90] have shown good correlation of total phenolic content with the oxidative stability of virgin olive oils. Baldioli et al. [91] have demonstrated that phenolic compounds are more effective than tocopherols in enhancing the stability of olive oils towards oxidation. Furthermore, there are some other nutrients in the Ginkgo leaves. Wong et al. [64] reported the discovery, synthesis, and characterization of a family of approximately 2 kilodaltons of super disulfide bond-restrained peptides-ginkgo peptides (*β-gB1* and *β-gB2*)—from plants of the Ginkgo genus, which have rarely been reported in both animals and plants.

#### 3.3.6. Fatty Acid Contents

Lipid oxidation primarily involves the degradation of unsaturated fatty acids (UFAs), with a saturation degree that critically influences thermal stability. Polyunsaturated fatty acids (PUFAs) exhibit increased susceptibility to oxidative degradation, generating aliphatic compounds [92,93]. Comparative analysis of flaxseed and soybean oils revealed distinct UFA profiles (Table 4 and Table 5): flaxseed oil was rich in linolenic acid (C18:3), whereas soybean oil was rich in linoleic acid (C18:2). The initial UFA content (C18:1, C18:2, and C18:3) was higher in soybean oil (87.35%) than in flaxseed oil (83.15%). During frying, both oils exhibit progressive UFA depletion and saturated fatty acid (SFA) accumulation. Palmitic acid (C16:0) demonstrated compositional stability among the SFAs, whereas UFAs (C18:2, C18:3) degraded most rapidly, particularly in flaxseed oil.

Treatment with GBLF mitigated SFA formation and UFA degradation more effectively than TBHQ. For instance, in flaxseed oil, palmitic acid increased by 17.21% with flavonoid treatment versus 22.81% with TBHQ, whereas linolenic acid decreased by 36.01% and 38.73%, respectively. Similar trends were observed in soybean oil: palmitic acid increased 1.57-fold (flavonoids) versus 1.63-fold (TBHQ), and linoleic acid degradation rates were 32.70% and 37.10%, respectively. These findings suggest that TBHQ has an inferior efficacy at high temperatures.

The oxidative instability of UFAs was correlated with reduced frying stability, which is consistent with previous studies [94,95]. For example, Jiang et al. [96] documented a linoleic acid reduction from 50.86% to 46.17% after 28 h of frying. *Ginkgo biloba* flavonoids effectively decelerate SFA accumulation and UFA oxidation, aligning with antioxidant mechanisms reported for rosemary and apple pomace extracts [93,97]. This underscores the role of natural antioxidants in enhancing the lipid oxidative stability during thermal processing. *Ginkgo biloba* extract improves nutrient utilization in broilers in a dose-dependent manner [56]. It is possible to use *EGb 761* of natural origin in medicine (treating diseases of the nervous system and cancer) [98].

## 4. Conclusions

This study has confirmed the feasibility of using GBLF as a natural and equally effective alternative to TBHQ in industrial applications [99,100,101,102,103,104,105,106,107,108], especially in the field of oil stabilization for food products, while considering both safety and functionality. In flaxseed oil and soybean oil, ultrasound-assisted GBLF extracts demonstrated oxidative protection, significantly reducing primary (33.79% and 17.71% lower peroxide value) and secondary (8.42% and 5.58% reduced anisidine values) oxidation markers, along with 18.4% and 17.35% lower acid value and 52.03% and 61.34% lower polar compound formation after 6 frying days. Furthermore, GBLF enhanced bioactive retention by mitigating tocopherol degradation compared with TBHQ, thereby preserving the nutritional quality of flaxseed and soybean oils. During the frying period, both oils exhibited a constant decrease in unsaturated fatty acids (C18:1, C18:2, and C18:3) and an increase in saturated fatty acids (C16:0 and C18:0), and GBLF showed more effective mitigation of unsaturated fatty acid reduction during frying.

The thermal stability of GBLF, driven by phenolic hydroxyl-mediated radical scavenging, effectively terminated lipid peroxidation chain reactions (LOO/LO) during deep frying, while dual-action mechanisms combining dominant free radical quenching and auxiliary metal chelation (Fe^2+^/Cu^+^) provided multi-target protection. To expedite commercial adoption, future work should prioritize long-term stability profiling under varied storage conditions (6–12 months), synergistic formulations with antioxidants such as rosemary extract, and process trials evaluating sensory and operational compatibility.

## Figures and Tables

**Table 1:** Experimental and predicted values of extraction efficiency in the Box-Behnken design.

Factor	Coded Symbol		Level	
		−1	0	1
Solvent: material ratio	X_1_	1:5	1:10	1:15
Ethanol volume fraction	X_2_	60%	70%	80%
Extraction temperature	X_3_	30 °C	40 °C	50 °C
Ultrasonic time	X_4_	80 s	100 s	120 s

**Table 2:** The data of experimental design.

Run	Solvent: Material Ratio (mL/g) X_1_	Ethanol Volume Fraction (%) X_2_	Extraction Temperature (°C) X_3_	Ultrasonic Time (s) X_4_	Extraction Rate of Flavonoids (%) Y
1	−1	−1	0	0	5.11
2	1	−1	0	0	5.60
3	−1	1	0	0	5.71
4	1	1	0	0	6.51
5	0	0	−1	−1	5.05
6	0	0	1	−1	5.66
7	0	0	−1	1	5.75
8	0	0	1	1	6.76
9	−1	0	0	−1	5.42
10	1	0	0	−1	5.61
11	−1	0	0	1	5.63
12	1	0	0	1	6.75
13	0	−1	−1	0	5.14
14	0	1	−1	0	5.70
15	0	−1	1	0	5.56
16	0	1	1	0	6.59
17	−1	0	−1	0	5.11
18	1	0	−1	0	5.68
19	−1	0	1	0	5.64
20	1	0	1	0	6.39
21	0	−1	0	−1	4.94
22	0	1	0	−1	5.48
23	0	−1	0	1	5.68
24	0	1	0	1	6.44
25	0	0	0	0	5.63
26	0	0	0	0	5.61
27	0	0	0	0	5.55
28	0	0	0	0	5.50
29	0	0	0	0	5.61

**Table 3:** Analysis of variance (ANOVA) of the reduced quadratic model of total flavonoid content as a function of ultrasonic extraction parameters.

Source	Sum of Squares	df	df	Mean Square	F-Value	Prob > F
Model	6.81	14	14	0.49	46.69	<0.0001
X_1_	1.29	1	1	1.29	123.55	<0.0001
X_2_	1.61	1	1	1.61	155.01	<0.0001
X_3_	1.44	1	1	1.44	137.77	<0.0001
X_4_	1.96	1	1	1.96	188.48	<0.0001
X_1_X_4_	0.21	1	1	0.21	20.49	0.0005
X_2_X_3_	0.056	1	1	0.056	5.41	0.0355
X_1_^2^	0.076	1	1	0.076	7.34	0.0170
X_3_^2^	0.056	1	1	0.056	5.36	0.0362
X_4_^2^	0.079	1	1	0.079	7.54	0.0158
Lack of Fit	0.13	10	10	0.013	4.84	0.0710

**Table 4:** Fatty acid content in GBLF with increasing frying time in flaxseed oil (%).

Fatty Acids	Flaxseed Oil	C16:0	C18:0	C18:1	C18:2	C18:3
0 days	TBHQ	5.48 ± 0.03 d	3.41 ± 0.01 f	18.18 ± 0.12 a	16.48 ± 0.24 a	48.49 ± 0.13 a
	GBLF	5.87 ± 0.06 d	4.05 ± 0.10 f	20.64 ± 0.56 a	16.57 ± 0.35 a	51.29 ± 0.51 a
1 days	TBHQ	5.53 ± 0.05 d	4.11 ± 0.11 e	16.62 ± 0.23 b	14.41 ± 0.32 b	46.57 ± 0.33 b
	GBLF	5.88 ± 0.05 d	4.54 ± 0.07 e	18.37 ± 0.34 b	14.33 ± 0.29 b	49.76 ± 0.60 b
2 days	TBHQ	5.86 ± 0.09 c	4.30 ± 0.13 d	14.75 ± 0.17 c	14.16 ± 0.16 b	42.64 ± 0.26 c
	GBLF	5.87 ± 0.06 d	4.74 ± 0.08 cd	17.26 ± 0.18 c	14.18 ± 0.33 b	43.42 ± 0.47 c
3 days	TBHQ	6.14 ± 0.11 bc	4.41 ± 0.06 cd	14.58 ± 0.38 c	13.45 ± 0.38 c	42.36 ± 0.55 c
	GBLF	6.00 ± 0.05 d	4.66 ± 0.08 de	16.45 ± 0.41 d	13.41 ± 0.33 c	41.19 ± 0.42 d
4 days	TBHQ	6.25 ± 0.26 b	4.54 ± 0.07 c	14.58 ± 0.39 c	13.34 ± 0.49 cd	40.77 ± 0.59 d
	GBLF	6.24 ± 0.06 c	4.87 ± 0.06 bc	14.52 ± 0.40 e	13.37 ± 0.46 c	41.16 ± 0.22 d
5 days	TBHQ	6.26 ± 0.26 b	4.94 ± 0.14 b	14.43 ± 0.36 c	12.85 ± 0.26 d	39.86 ± 1.17 d
	GBLF	6.45 ± 0.12 b	4.94 ± 0.09 b	14.15 ± 0.74 e	12.54 ± 0.42 d	40.22 ± 0.30 e
6 days	TBHQ	6.73 ± 0.23 a	5.18 ± 0.05 a	11.84 ± 0.28 d	10.33 ± 0.30 e	29.71 ± 0.61 e
	GBLF	6.88 ± 0.11 a	5.37 ± 0.07 a	12.56 ± 0.42 f	10.38 ± 0.34 e	32.82 ± 0.75 f

Values are expressed as mean ± standard deviation (SD) of triplicate measurements. Different lowercase superscript letters (a, b, c, etc.) within the same column indicate statistically significant differences between groups according to Duncan’s multiple range test at *p* < 0.05.

**Table 5:** Fatty acid content in GBLF with increasing frying time in soybean oil (%).

Fatty Acids	Soybean Oil	C16:0	C18:0	C18:1	C18:2	C18:3
0 days	TBHQ	10.45 ± 0.51 g	4.07 ± 0.07 c	29.62 ± 0.56 a	51.45 ± 0.90 a	6.28 ± 0.25 a
	GBLF	11.06 ± 0.45 g	4.85 ± 0.09 cd	30.23 ± 0.55 a	50.88 ± 0.38 a	6.85 ± 0.09 a
1 days	TBHQ	12.70 ± 0.40 f	4.07 ± 0.08 c	28.67 ± 0.33 b	50.76 ± 0.46 ab	5.84 ± 0.11 b
	GBLF	13.38 ± 0.31 f	4.82 ± 0.15 cd	29.76 ± 0.34 a	50.78 ± 0.81 a	6.04 ± 0.10 b
2 days	TBHQ	16.55 ± 0.32 e	4.13 ± 0.07 bc	27.10 ± 0.35 c	49.65 ± 0.68 b	5.43 ± 0.12 c
	GBLF	18.28 ± 0.32 e	4.83 ± 0.10 cd	28.55 ± 0.34 b	50.75 ± 0.56 a	5.84 ± 0.07 b
3 days	TBHQ	20.49 ± 0.44 d	4.22 ± 0.10 abc	25.77 ± 0.20 d	47.46 ± 0.51 c	4.85 ± 0.12 d
	GBLF	22.32 ± 0.40 d	4.71 ± 0.14 d	25.95 ± 0.16 c	48.59 ± 0.96 b	5.03 ± 0.14 c
4 days	TBHQ	22.24 ± 0.60 c	4.30 ± 0.17 ab	26.64 ± 0.31 e	45.47 ± 0.64 d	4.39 ± 0.21 e
	GBLF	25.58 ± 0.44 c	5.02 ± 0.13 bc	25.22 ± 0.19 d	46.60 ± 0.42 c	4.94 ± 0.10 c
5 days	TBHQ	24.31 ± 0.64 b	4.41 ± 0.12 a	23.79 ± 0.33 f	40.28 ± 0.70 e	3.80 ± 0.13 f
	GBLF	26.25 ± 0.28 b	5.29 ± 0.15 ab	23.81 ± 0.22 e	41.11 ± 0.38 d	4.36 ± 0.42 d
6 days	TBHQ	27.48 ± 0.36 a	4.31 ± 0.18 ab	23.35 ± 0.37 f	32.36 ± 0.53 f	2.76 ± 0.12 g
	GBLF	28.45 ± 0.39 a	5.42 ± 0.26 a	23.89 ± 0.23 e	34.24 ± 0.54 e	2.90 ± 0.18 e

Values are expressed as mean ± standard deviation (SD) of triplicate measurements. Different lowercase superscript letters (a, b, c, etc.) within the same column indicate statistically significant differences between groups according to Duncan’s multiple range test at *p* < 0.05.

## Data Availability

The original contributions presented in this study are included in the article. Further inquiries can be directed to the corresponding author.
